# *Sinobdellalongitubulus*, a new species of spiny eel (Pisces, Mastacembelidae) from the Zhu-Jiang Basin, with a note on the type locality of *S.sinensis* (Bleeker, 1870)

**DOI:** 10.3897/BDJ.12.e123990

**Published:** 2024-06-19

**Authors:** Peng Shan, Guangyu Li, E Zhang

**Affiliations:** 1 Huazhong Agricultural University, Wuhan, China Huazhong Agricultural University Wuhan China; 2 Institute of Hydrobiology, Chinese Academy of Sciences, Wuhan, China Institute of Hydrobiology, Chinese Academy of Sciences Wuhan China

**Keywords:** *Sinobdella*, new species, taxonomy, Zhu-Jiang Basin

## Abstract

**Background:**

The spiny eel genus *Sinobdella* belongs to the family Mastacembelidae of the order Synbranchiformes. Kottelat and Lim (1994) utilised *Rhynchobdellasinensis* as the type species to propose the genus. Currently, it contains a single species widespread in eastern and southern China and northern Vietnam.

**New information:**

*Sinobdellalongitubulus*, a new species of spiny eel, is here described from the Xi-Jiang of the Zhu-Jiang Basin in Guangxi Zhuang Autonomous Region, southern China. It differs from the single congeneric species *S.sinensis* in having a more or less white-brown reticulated pattern on the flank, two tubular anterior nostrils longer than or equal to the rostral appendage, an anal fin heavily mottled with dark brown markings and white spots and bearing a narrow white distal margin; shorter pre-anal length; and fewer abdominal vertebrae. The validity of this new species is corroborated by its monophyly recovered in a COI gene-based phylogenetic analysis and its significant sequence divergence with *S.sinensis*. A note on the type locality of *S.sinensis* is also given; its type specimen is possibly from mountain streams of Jiangxi Province, in the lower Chang-Jiang Basin.

## Introduction

The family Mastacembelidae, widely known as spiny eels, consists of anguilliform percomorph fishes, characterised by continuous dorsal spines along the mid-line dorsal body. These species are extensively distributed in tropical Africa, the Middle East and South and Southeast Asia, where they inhabit diverse freshwater environments such as lakes, streams and rivers, feeding on zooplankton, aquatic insect larvae and small fish (Nelson et al. 2016). Amongst three genera to date identified in the family (Roberts 1986, Vreven and Teugels 2005), *Sinobdella* is the only monotypic genus spanning from the Liao-He, an independent river flowing into Bohai Sea, in Liaoning Province (Xie 2007), through eastern and southern China (including Taiwan Island) (Shao 2022), to the Red River in northern Vietnam (Kottelat 2001, Kottelat 2013).

The generic placement and specific status of the single species *S.sinensis* (Bleeker 1870a) have ever remained contentious. It was initially described in *Rhynchobdella* Bloch & Schneider and later referred to either *Mastacembelus* Scopoli (Wang 1984, Wang et al. 2001) or *Pararhynchobdella* Bleeker (Zhu 1995). This spiny eel belongs to its own genus (Kottelat and Lim 1994). In Chinese literature , this species had been synonymised with *Mastacembelusaculeatus* (Basilewsky, 1855) before it was viewed as valid (Zheng 1989, Liu 1991, Zhou 1997, Tang et al. 2001).

The taxonomy of *S.sinensis* is still poorly understood due to a lack of information on its precise type locality. The original description stated that this species was from China, but without specifying its accurate location (Bleeker 1870a). This poses a hindrance to in-depth taxonomic investigations on the species diversity of *S.sinensis*, so leading to a taxonomic inertia of this spiny eel since its original description. It has widely been regarded as a widespread species in China (Zhang et al. 2016). However, our ongoing taxonomic study of this species from China demonstrates significant morphological and genetic differentiations between the Pearl River (= Zhu-Jiang in mainland China) and Yangtze River (= Chang-Jiang) Basin populations. Our textual research revealed that its type locality is mountain streams of Jiangxi Province, in the lower Chang-Jiang Basin (see explanation in the Discussion section). Clearly, the true *S.sinensis* is represented by the Chang-Jiang Basin population. Many specimens, caught during 2021 to 2023 by us from the Meng-Jiang (in Pingnan and Mengshan Counties) and Hongshui-He (in Shanglin County) of the Zhu-Jiang Basin in China, belong to an unidentified recognised species. The objectives of the present study are to: (1) provide a detailed description of this unidentified species and (2) clarify the uncertainty concerning the type locality of *S.sinensis*.

## Materials and methods

### Specimen sampling, preservation and curation

The specimens of *Sinobdella* used in this study were obtained via field sampling, in accordance with the Chinese Laboratory Animal Welfare and Ethics animal welfare laws (GB/T35892–2018). Some caught individuals after being anaesthetised were stored in 10% formalin for morphological observation and permanent curation, following the removal of a small flip of pectoral fin preserved in 95% ethanol. Other individuals were directly preserved in 95% ethanol or 10% formalin after being anaesthetised. Examined specimens are housed in the Museum of the Institute of Hydrobiology (IHB), Chinese Academy of Sciences, Wuhan City, Hubei Province, P. R. China, British Museum of Natural History (BMNH) and the Muséum national d'Histoire naturelle (MNHN).

### Morphological analysis

Measurements were taken point to point with a digital caliper linked directly to a data-recording computer and data recorded to the nearest 0.1 mm. Measurements were made on the left side of specimens whenever possible. The counts of vertebra, dorsal spine and dorsal- and anal-fin rays were taken from X-ray photographs. Pectoral- and caudal-fin rays are difficult to count in a dissecting scope and, thus, not included here. All counts and measurements follow Vreven and Teugels (1996). If the number of fin rays and vertebrae could not be counted in a given X-ray photograph, the part of this photograph will be magnified until it can be clearly counted. Measurements of head were expressed as percentages of lateral head length (HL) and HL and measurements of other parts of body were given as proportions of standard length (SL). GraphPad Prism 9 (GraphPad Prism Inc.) was utilised for the basic statistical analysis of morphometric measurements.

### Molecular analysis

Total genomic DNA was extracted from the pectoral fin stored in 95% ethanol using the TIANamp Genomic DNA Kit (Tiangen Biotech Co., Ltd, Beijing, China) following the manufacturer's recommendations. The mitochondrial cytochrome C oxidase subunit I (COI) gene was chosen for phylogenetic analysis. Amplification and sequencing of the target gene were achieved using the COI-F1 primer (5′GTGGCAATCACACGTTGAT′3) (Jiang 2018), along with a primer specifically designed for this study, COI-R1 (5′ATGGAGGTTCGATTCCTTC′3). The target gene was amplified by polymerase chain reaction (PCR) in a 25 μl mixture, consisting of 1 μl of each primer, 12.5 μl of Taq Master mix (Genesand Biotech Co. Ltd., Beijing, China), 1 μl of template DNA and 9.5 μl of double-distilled water (ddH_2_O). The PCR was executed under the following thermocycling conditions: an initial denaturation at 94°C for 3 minutes, followed by 35 cycles of denaturation at 94°C for 45 seconds, annealing at 53°C for 40 seconds and extension at 72°C for 1 minute and 30 seconds, with final extension at 72°C for 8 minutes. The PCR products were subsequently stored at 4°C. Sequencing was performed by Aokedingsheng Biotechnology Company (Wuhan, China) and the sequences obtained in this study were submitted to GenBank.

### Phylogenetic analysis

The amplified 136 gene sequences were used for molecular phylogenetic analysis along with other GenBank-retrieved sequences of the same gene from four outgroup species: *Monopterusalbus* (KP779625), *Mastacembelusarmatus* (KY609156), *M.favus* (OR804504.1) and *M.erythrotaenia* (NC_035141.1). Raw sequences were edited using Seqman in DNAstar (DNAStar Inc., Madison, WI, USA) and aligned using Seaview v.4.2.5 (Gouy et al. 2010). DnaSP v.6 (Rozas et al. 2017) was utilised for genetic diversity analyses and haplotype filtering. Haplotype sequences were used for phylogenetic analyses. PhyloSuite (Zhang et al. 2019) was employed for phylogenetic analyses. The best-fit model of nucleotide evolution, based on Akaike’s Information Criterion, was selected using ModelFinder (Kalyaanamoorthy et al. 2017). MrBayes v.3.2.6 (Ronquist et al. 2012) was utilised for building the Bayesian Inference (BI) tree with the selected model: GTR+I+G+F, applying the optimal nucleotide evolution model and the MCMC method with four chains running simultaneously for 10,000,000 generations to calculate posterior probability. The initial 25% of sampled tree were discarded as burn-in. Maximum Likelihood phylogenies were inferred using IQ-TREE (Nguyen et al. 2014) under the TIM2+I+G4+F model with 5000 ultrafast (Minh et al. 2013) bootstraps. The phylogenetic tree was edited in FigTree v.1.4.3 (Rambaut 2018). In addition, uncorrected genetic distances (p-distances) were calculated using MEGA v.11.0 (Tamura et al. 2021).

## Data resources

All the sequences in this study were retrieved from GenBank and the accession numbers of the newly-determined sequences in this study are shown in Suppl. material 1.

## Taxon treatments

### Sinobdella longitubulus

Shan & Zhang 2024 sp. nov.

916F5A0E-328C-5EF9-BBAE-43EA7C93F09D

urn:lsid:zoobank.org:pub:B38159CC-3780-4F45-9A03-AE45C1213101

#### Materials

- **Type status:** Holotype. **Occurrence:** recordNumber: IHB 202303066738; individualCount: 1; occurrenceID: 0A39F7E5-D24D-5E38-B46B-DACB7D000E48; **Taxon:** scientificName: *Sinobdellalongitubulus*; kingdom: Animalia; phylum: Chordata; class: Actinopterygii; order: Synbranchiformes; family: Mastacembelidae; genus: Sinobdella; **Location:** waterBody: Zhu-Jiang Basin; country: China; stateProvince: Guangxi; county: Pingnan; municipality: Lilia Village; locality: Datong-Jiang, a tributary to Meng-Jiang of Xi-Jiang; verbatimElevation: 135 m; verbatimCoordinates: 23°57′36″N 110°18′55″E; verbatimSRS: WGS84; **Identification:** identifiedBy: Peng Shan; dateIdentified: 2023; **Record Level:** collectionCode: Fish; basisOfRecord: PreservedSpecimen
- **Type status:** Paratype. **Occurrence:** recordNumber: IHB 202303066739-40; individualCount: 2; occurrenceID: C43F36CB-B23A-5897-B277-8003717129C2; **Taxon:** scientificName: *Sinobdellalongitubulus*; kingdom: Animalia; phylum: Chordata; class: Actinopterygii; order: Synbranchiformes; family: Mastacembelidae; genus: Sinobdella; **Location:** waterBody: Zhu-Jiang Basin; country: China; stateProvince: Guangxi; county: Pingnan; municipality: Lilia Village; locality: Datong-Jiang, a tributary to Meng-Jiang of Xi-Jiang; verbatimElevation: 135 m; verbatimCoordinates: 23°57′36″N 110°18′55″E; verbatimSRS: WGS84; **Identification:** identifiedBy: Peng Shan; dateIdentified: 2023; **Record Level:** collectionCode: Fish; basisOfRecord: PreservedSpecimen
- **Type status:** Paratype. **Occurrence:** recordNumber: IHB 202104052032,3705; individualCount: 2; occurrenceID: 150682DC-8100-50B1-B46E-987F6287A81B; **Taxon:** scientificName: *Sinobdellalongitubulus*; kingdom: Animalia; phylum: Chordata; class: Actinopterygii; order: Synbranchiformes; family: Mastacembelidae; genus: Sinobdella; **Location:** waterBody: Zhu-Jiang Basin; country: China; stateProvince: Guangxi; county: Mengshan; municipality: Donghu Village; locality: Changping-He discharging into Meng-Jiang of Xi-Jiang; verbatimElevation: 258 m; verbatimCoordinates: 24°17′16″N 110°32′35″E; verbatimSRS: WGS84; **Identification:** identifiedBy: Peng Shan; dateIdentified: 2021; **Record Level:** collectionCode: Fish; basisOfRecord: PreservedSpecimen
- **Type status:** Paratype. **Occurrence:** recordNumber: IHB 202310064917-28; individualCount: 12; occurrenceID: E93A45AA-DC35-5C67-8C42-761BE6CED7F7; **Taxon:** scientificName: *Sinobdellalongitubulus*; kingdom: Animalia; phylum: Chordata; class: Actinopterygii; order: Synbranchiformes; family: Mastacembelidae; genus: Sinobdella; **Location:** waterBody: Zhu-Jiang Basin; country: China; stateProvince: Guangxi; county: Shanglin; locality: Qingshui-He flowing into Hongshui-He of Xi-Jiang; verbatimCoordinates: 23°33′14″N 108°25′16″E; verbatimSRS: WGS84; **Identification:** identifiedBy: Peng Shan; dateIdentified: 2023; **Record Level:** collectionCode: Fish; basisOfRecord: PreservedSpecimen
- **Type status:** Other material. **Occurrence:** recordNumber: IHB 202206063298-3302; individualCount: 5; occurrenceID: 7097B098-CFD4-5538-9057-F211F9458F3C; **Taxon:** scientificName: *Sinobdellasinensis*; acceptedNameUsage: *Sinobdellasinensis* Bleeker,1870; kingdom: Animalia; phylum: Chordata; class: Actinopterygii; order: Synbranchiformes; family: Mastacembelidae; genus: Sinobdella; **Location:** waterBody: lower Chang-Jiang basin; country: China; stateProvince: Anhui; county: Susong; locality: lower Chang-Jiang; verbatimCoordinates: 30°11′50″N 116°4′37″E; verbatimSRS: WGS84; **Identification:** identifiedBy: Peng Shan; dateIdentified: 2022; **Record Level:** collectionCode: Fish; basisOfRecord: PreservedSpecimen
- **Type status:** Other material. **Occurrence:** recordNumber: IHB 202204062039-44; individualCount: 5; occurrenceID: 941804DF-F1BD-5DFA-9ECD-43FAA9E19780; **Taxon:** scientificName: *Sinobdellasinensis*; acceptedNameUsage: *Sinobdellasinensis* Bleeker,1870; kingdom: Animalia; phylum: Chordata; class: Actinopterygii; order: Synbranchiformes; family: Mastacembelidae; genus: Sinobdella; **Location:** waterBody: middle Chang-Jiang basin; country: China; stateProvince: Hubei; municipality: Xiangyang City; locality: Han-Jiang of middle Chang-Jiang; verbatimCoordinates: 32°12′57″N 112°21′42″E; verbatimSRS: WGS84; **Identification:** identifiedBy: Peng Shan; dateIdentified: 2022; **Record Level:** collectionCode: Fish; basisOfRecord: PreservedSpecimen
- **Type status:** Other material. **Occurrence:** recordNumber: IHB 202203061530-34; individualCount: 4; occurrenceID: 15C38902-CB39-5795-B5DB-F4432C0A1DA4; **Taxon:** scientificName: *Sinobdellasinensis*; acceptedNameUsage: *Sinobdellasinensis* Bleeker,1870; kingdom: Animalia; phylum: Chordata; class: Actinopterygii; order: Synbranchiformes; family: Mastacembelidae; genus: Sinobdella; **Location:** waterBody: Luan-He; country: China; stateProvince: Hebei; county: Luanzhou; locality: Luan-He (an independent river flowing into Bohai Sea); verbatimCoordinates: 39°42′29″N 118°46′10″E; verbatimSRS: WGS84; **Identification:** identifiedBy: Peng Shan; dateIdentified: 2022; **Record Level:** collectionCode: Fish; basisOfRecord: PreservedSpecimen
- **Type status:** Other material. **Occurrence:** recordNumber: IHB 202203061535-38; individualCount: 4; occurrenceID: CE23B704-CB7E-52C9-BA50-1775B6376F6A; **Taxon:** scientificName: *Sinobdellasinensis*; acceptedNameUsage: *Sinobdellasinensis* Bleeker,1870; kingdom: Animalia; phylum: Chordata; class: Actinopterygii; order: Synbranchiformes; family: Mastacembelidae; genus: Sinobdella; **Location:** waterBody: Qiantang-Jiang basin; country: China; stateProvince: Zhejiang; county: Changshan; municipality: Quzhou City; locality: Changshangang; verbatimCoordinates: 28°56′15″N 118°28′15″E; verbatimSRS: WGS84; **Identification:** identifiedBy: Peng Shan; dateIdentified: 2022; **Record Level:** collectionCode: Fish; basisOfRecord: PreservedSpecimen
- **Type status:** Other material. **Occurrence:** recordNumber: IHB 202209061967; individualCount: 1; occurrenceID: 4B8DDD23-630C-53C5-939B-CDF9456D2C2E; **Taxon:** scientificName: *Sinobdellasinensis*; acceptedNameUsage: *Sinobdellasinensis* Bleeker,1870; kingdom: Animalia; phylum: Chordata; class: Actinopterygii; order: Synbranchiformes; family: Mastacembelidae; genus: Sinobdella; **Location:** waterBody: Min-Jiang basin; country: China; stateProvince: Fujian; county: Sanming; locality: Min-Jiang; verbatimCoordinates: 26°14′52″N 117°32′43″E; verbatimSRS: WGS84; **Identification:** identifiedBy: Peng Shan; dateIdentified: 2022; **Record Level:** collectionCode: Fish; basisOfRecord: PreservedSpecimen
- **Type status:** Other material. **Occurrence:** recordNumber: IHB 202010051336-37, IHB 202203064318; individualCount: 3; occurrenceID: B63617B7-0495-5B97-B739-A4E0669AAA1B; **Taxon:** scientificName: *Sinobdellasinensis*; acceptedNameUsage: *Sinobdellasinensis* Bleeker,1870; kingdom: Animalia; phylum: Chordata; class: Actinopterygii; order: Synbranchiformes; family: Mastacembelidae; genus: Sinobdella; **Location:** waterBody: Zhu-Jiang Basin; country: China; stateProvince: Guangxi; county: Yongfu; locality: Luoqing-Jiang of Xi-Jiang; verbatimCoordinates: 25°5′55″N 109°55′44″E; verbatimSRS: WGS84; **Identification:** identifiedBy: Peng Shan; dateIdentified: 2023; **Record Level:** collectionCode: Fish; basisOfRecord: PreservedSpecimen
- **Type status:** Other material. **Occurrence:** recordNumber: IHB 202306064822; individualCount: 1; occurrenceID: AA2E9ED2-D736-5359-AD9E-35D17563AD6F; **Taxon:** scientificName: *Sinobdellasinensis*; acceptedNameUsage: *Sinobdellasinensis* Bleeker,1870; kingdom: Animalia; phylum: Chordata; class: Actinopterygii; order: Synbranchiformes; family: Mastacembelidae; genus: Sinobdella; **Location:** waterBody: Chang-Jiang basin; country: China; stateProvince: Guangxi; county: Quanzhou; locality: Xiang-Jiang of Chang-Jiang basin; verbatimCoordinates: 25°52′51″N 110°58′56″E; verbatimSRS: WGS84; **Identification:** identifiedBy: Peng Shan; dateIdentified: 2023; **Record Level:** collectionCode: Fish; basisOfRecord: PreservedSpecimen
- **Type status:** Other material. **Occurrence:** recordNumber: IHB 202306064812, 4820; individualCount: 2; occurrenceID: 2AD5A3E4-41B3-53FE-BD6E-8ACFCCDEA307; **Taxon:** scientificName: *Sinobdellasinensis*; acceptedNameUsage: *Sinobdellasinensis* Bleeker,1870; kingdom: Animalia; phylum: Chordata; class: Actinopterygii; order: Synbranchiformes; family: Mastacembelidae; genus: Sinobdella; **Location:** waterBody: Huai-He basin; country: China; stateProvince: Henan; county: Shangcai; locality: Hong-He of Huai-He basin; verbatimCoordinates: 33°22′58″N 114°14′20″E; verbatimSRS: WGS84; **Identification:** identifiedBy: Peng Shan; dateIdentified: 2023

#### Description

**Figure 1.**
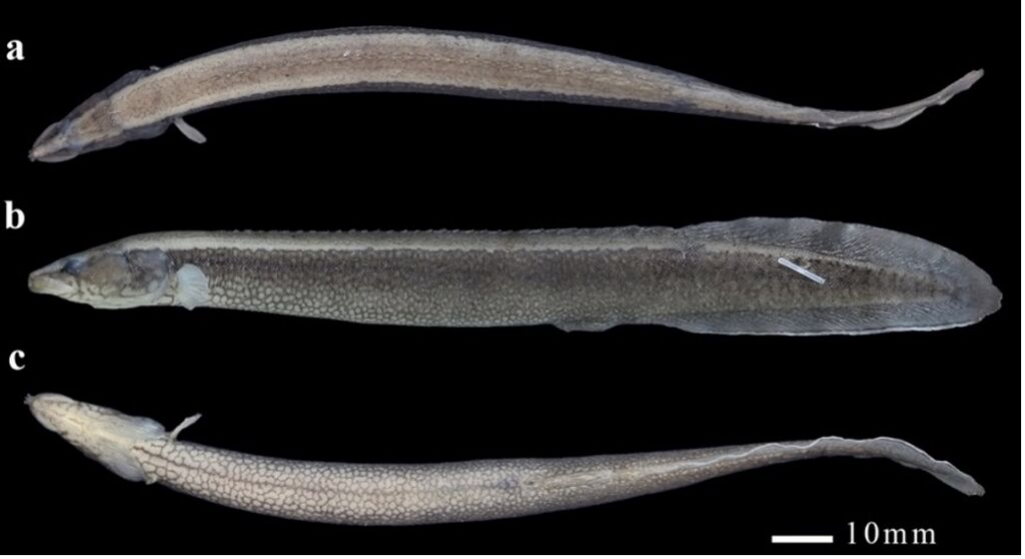
*S.longitubulus*, holotype, IHB 202303066738, 153.3 mm SL, dorsal (a), lateral (b) and ventral (c) views. China: Guangxi Province: Guigang City: Pingnan County: Lilia Village: Datong-Jiang, a stream tributary to Meng-Jiang flowing into Xi-Jiang of Zhu-Jiang basin.

Morphometric measurements and counts are presented in Table 1. See Figs 1, 2a for general body appearance.

Body elongated, oval in cross-section and compressed laterally in caudal region. Body depth evenly deep towards soft anal-fin origin, then gradually decreasing towards caudal-fin base, terminating in form of a pointed or rounded fin tail-like structure. Tail region relatively short. Pre-anal length slightly greater than postanal length. Body covered with tiny scales without lateral line.

Head short and pointed, with snout produced into fleshy and long rostral appendage projecting from upper jaw. Two tubular anterior nostrils located on underside of rostral appendage, longer than or equal to this appendage. Posterior nares oval, horizontal axis longest and situated in front of eyes. Eyes small, laterodorsally located at anterior half of head. Posteriorly growing single pre-orbital spine on right/left side of head, located under eyes and buried under skin, with ending point extending beyond anterior margin of eye, but not reaching middle of eye. Mouth inferior and horse-shoe-shaped. Lips thick and fleshy. Jaws with numerous small, pointed teeth; upper jaw longer than lower jaw. Angle of jaws between the posterior edge of the posterior external nare and the anterior edge of the eye. Preoperculum unarmed. Upper extremity of gill opening immediately above dorsal end of pectoral-fin base, slightly anterior to vertical through ventral end of pectoral-fin base.

Dorsal fin long and divided into two parts: dorsal spines and dorsal-fin. Dorsal spines 32 to 33, increasing in size from first to second last spine; last spine smaller than second last spine, situated anterior to base of first soft dorsal-fin ray. Soft dorsal-fin rays 50-60. Anal spines 3 and soft anal-fin rays 50-60; anterior end of dorsal spines posterior to pectoral-fin insertion and anterior end of soft dorsal-fin rays slightly posterior to anterior end of soft anal fin. Pectoral fins small, extended latero-posteriorly, with their bases located mainly below upper margins of gill openings. No pelvic fins. Origin of exposed anal spines slightly posterior to anal. Caudal fin very small and rounded with 7-8 rays, confluent with soft dorsal- and anal-fins. First and second anal spines supported by well-developed first anal spine pterygiophore and third anal spine derived from second spine pterygiophore, situated anteriorly to first soft anal-fin ray. Second anal spine placed close to first spine, but distinctly distant from third spine, much longer than both.

#### Diagnosis

**Figure 4.**
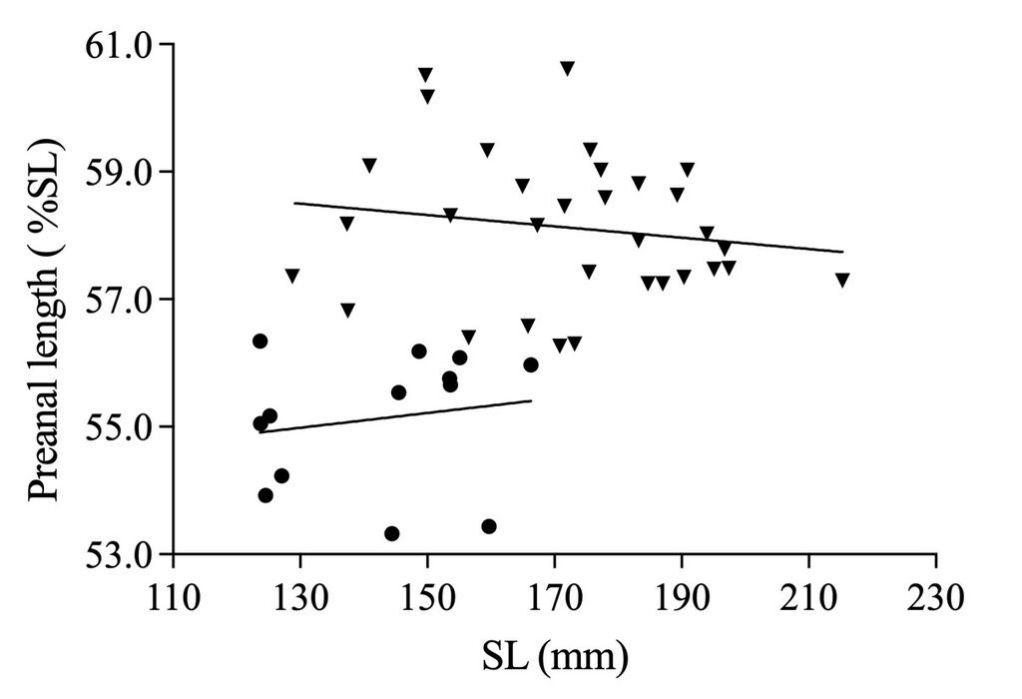
Relationship between pre-anal length (%SL) and SL for two species of *Sinobdellasinensis* (▼), y=-0,008887x+59.65 and *S.longitubulus* sp. nov. (●), y=0.01158x+53.48.

*Sinobdellalongitubulus* is clearly distinguished from the single congeneric species (*S.sinensis*) by having a more or less white-brown reticulated pattern (vs. many dark brown vertical bars, with very narrow light yellow interspaces) on the flank (Fig. 2), two tubular anterior nostrils longer than or equal to (vs. shorter than) the rostral appendage (Fig. 3), an anal fin heavily mottled with dark brown markings and white spots and bearing a narrow white distal margin (vs. black with a relatively wide light white distal margin) (Fig. 2); shorter pre-anal length (53.3-56.2 vs. 56.3-60.6 % SL; see Fig. 4) and fewer abdominal vertebrae (32-33, mean = 32.9 vs. 34-36, mean = 35.1) (Table 1).

#### Etymology

The epithet name, used here as a noun, is derived from the Latin word *longus* (= long) and *tubulus* (= pipe), alluding to two longer tubes modified from anterior nostrils. The common Chinese name here suggested for this new species is “长管华刺鳅”.

#### Distribution

**Figure 5.**
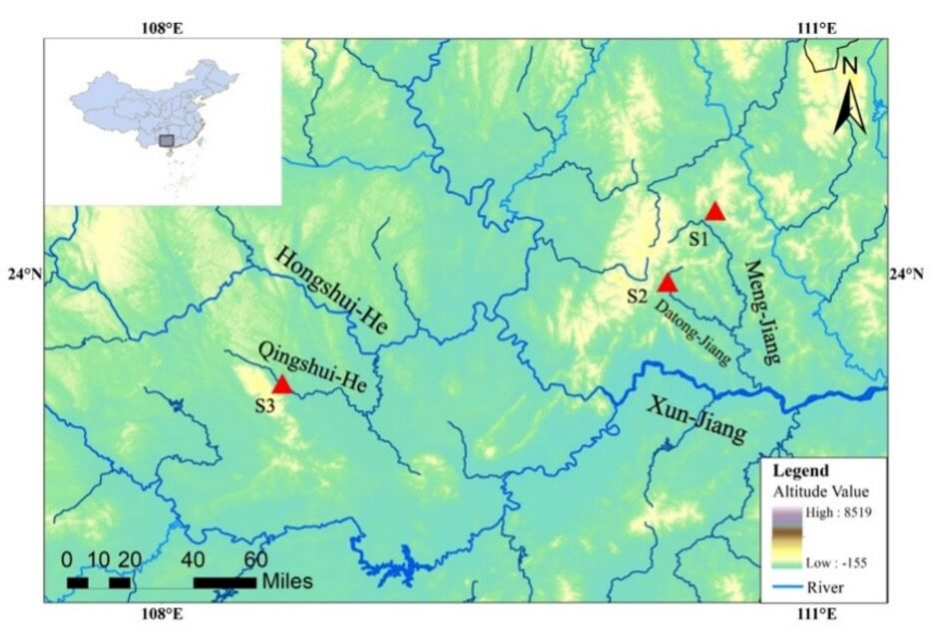
Map showing three sampling sites of the type specimens of *S.longitubulus*. S1, Donghu Village, Mengshan County, Wuzhou City; S2, Lilia Village, Pingnan County, Guigang City; and S3, Shanglin County, Nanning City, Guangxi Province, P. R. China.

**Figure 6.**
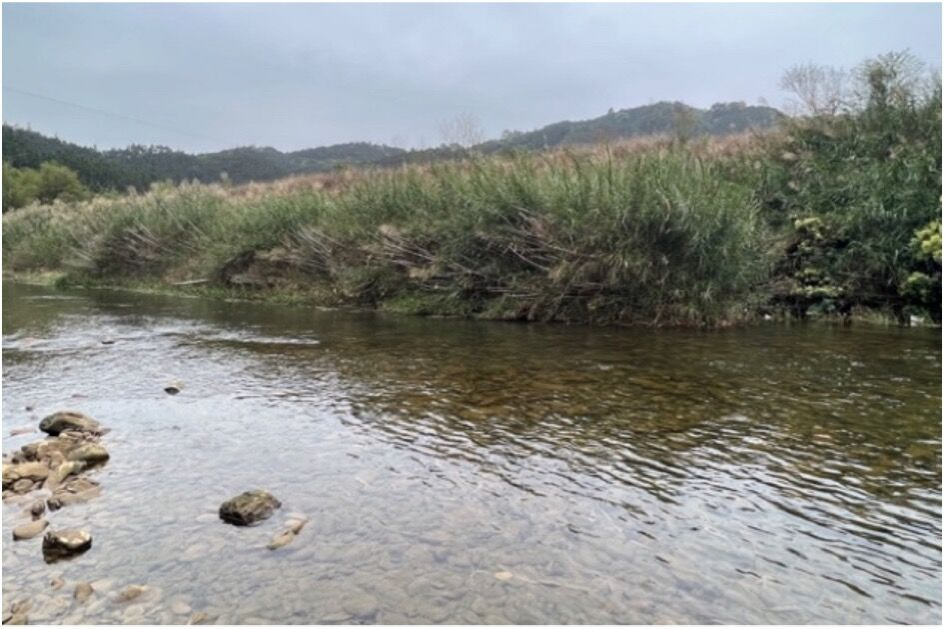
Habitat at the type locality of *Sinobdellalongitubulus* in the Datong-Jiang, a stream tributary to the Xun-Jiang of Zhu-Jiang Basin at Lilia Village, Pingnan County, Guangxi Province, China.

*Sinobdellalongitubulus* is currently known from the Meng-Jiang emptying into the Xi-Jiang and the Qingshui-He, a stream tributary to the Hongshui-He discharging into the Xi-Jiang of Zhu-Jiang Basin (Fig. 5). It was collected in shallow running waters with mixed bottom including pebbles, cobbles, gravel and boulders (Fig. 6). Co-existing species are: *Traccatichthyspulcher*, *Vanmaneniastenosoma*, *Opsariichthysacutipinnis*, *Acrossocheilusparallens*, *Rhinogobiusduospilus*, *Coreopercawhiteheadi* and *Schisturafasciolata*.

#### Colouration

In formalin-preserved specimens (Fig. 1), top of head whitish-brown and cheek and opercula brown, with a reticulated pattern of brown markings on ventral surface of head. A broad light yellowish mid-dorsal band extending from behind head to soft dorsal-fin origin and two narrow whitish stripes parallel to this band laterally, wider and lightened anteriorly and prolonging backwards under soft dorsal-fin base. Lateral body brown with numerous small white spots, more or less inconspicuous on upper two-thirds of abdomen and caudal region and conspicuous and restricted only to lower one-third of abdomen to form a white-brown reticulated pattern, continued by a broad extension across belly. Pectoral fin pale, dorsal and caudal fins dark grey and anal fin dark grey with a narrow whitish distal margin and a dark black submarginal stripe.

In freshly-、collected specimens (Fig. 2a), dorsum of head and body yellowish-brown from snout tip to origin of soft dorsal fin, with two black longitudinal zigzag lines parallel to dorsal mid-line on each side, more or less connected to constitute a reticulated pattern of markings. Lateral body dark brown with numerous irregular white spots scattering densely over abdomen and sparsely on caudal region, more or less distinct and restricted to lower half of flank to form a reticulated pattern of markings. Continued dorsal and caudal fins light yellowish-brown with an indistinct reticulated pattern. Pectoral fins yellowish or orange and anal fin heavily mottled with dark brown markings and white spots, bearing a narrow light white distal margin and a very narrow submarginal black stripe.

## Analysis

A total of 136 COI gene sequences of 1448 bp in length of two *Sinobdella* species were here amplified. Forty haplotypes (including four outgroups) were detected from the sequences (Suppl. material 1). These sequences were used for molecular phylogenetic analysis along with four sequences from the outgroups: *Monopterusalbus* (one), *Mastacembelusarmatus* (one), *M.favus* (one) and *M.erythrotaenia* (one). The sequences consisted of 969 conserved sites, 479 variable sites, 327 parsimony informative sites and 152 singleton sites. The nucleotide frequencies were 25.9% (A), 30.8% (T), 26.0% (C) and 17.2% (G).

**Figure 7.**
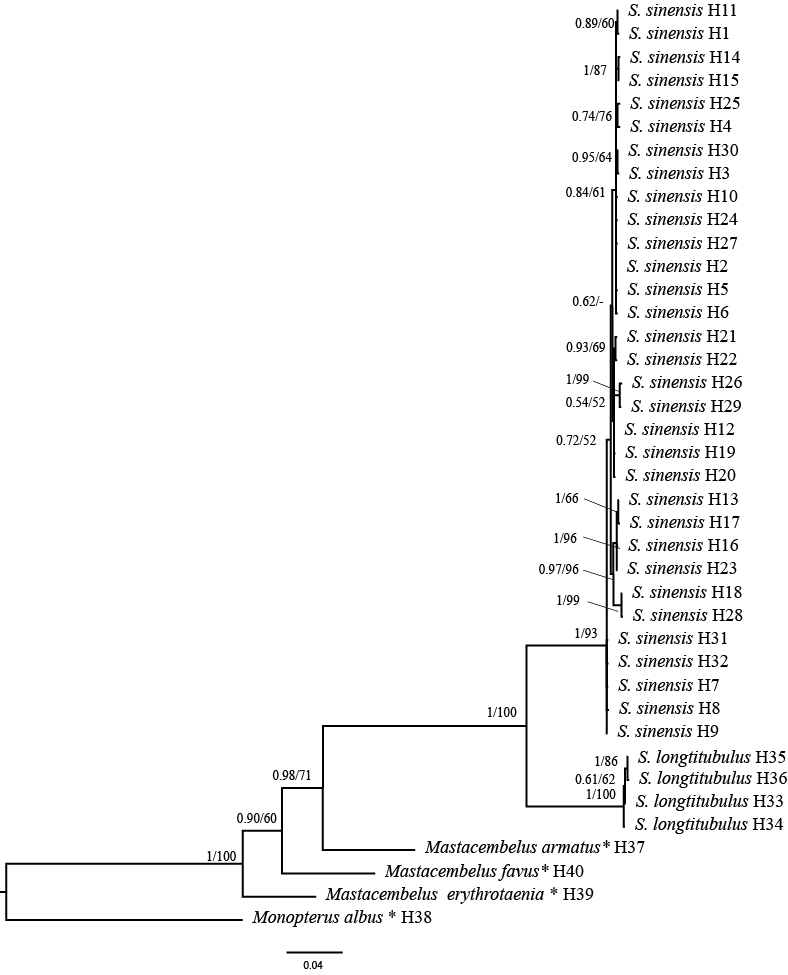
Bayesian Inference tree based on the COI gene for two species of *Sinobdella* and three species of *Mastacembelus*. Bayesian posterior probabilities (> 0.5), maximum Likelihood bootstrap values (> 50%) are shown, respectively. Dashes represent nodes with bootstrap support lower than 50%.

Given that Bayesian Inference (BI) and Maximum Likelihood (ML) analyses produced overall identical topologies, only the BI tree with Bayesian posterior probabilities (PP) and bootstrap support (BS) value were provided in Fig. 7. From the tree topologies, samples of *S.longitubulus* were strongly supported (PP = 1.0/BS = 100) to cluster into a lineage further constituting a well-supported (PP = 1.0/BS = 100) clade together with *S.sinensis*.

The genetic distances (p-distances) within and between species were calculated. Intraspecific genetic distance for sampled species of *S.sinensis* and *S.longitubulus* were 0.5% and 0.1%, respectively. Interspecific genetic distance between both were 10.5%.

## Discussion

Bleeker (1870a) described *Rhynchobdellasinensis*, based on a single 199 mm TL specimen caught in China. Despite no indication of the accurate type locality in the original description, Bleeker (1870b) noted that this species was one of 44 species then identified from Chinese specimens predominantly collected from the Yang-Tse-Kiang (or Yangtze River = Chang-Jiang in mainland China) and associated Lake Po Yang (= Lake Poyang) and Kan-Kiang River (presently Gan-Jiang, a stream of Lake Poyang) and the waters of Ning-Po (presently Ningbo). He further stated that these Chinese specimens, received from the administration of Jardin royal des plantes médicinales (the predecessor of the Muséum national d'Histoire naturelle, MNHN), constituted part of the collections by C.P. Dabry de Thiersant, G.E. Simon and A. David intended for the Museum in Paris (MNHN); the board of the Museum in Paris invited him to identify them. Apparently, the type specimen of *S.sinensis* ought to be housed at MNHN rather than at BMNH (British Museum of Natural History) and the collector of the type is likely one of the three persons mentioned.

There are seven records of Chinese spiny eels currently housed in MNHN (Table 2). Seven specimens [MNHN-IC-0000-5230 (two), 5573 (four) and 7490 (one)] of unknown source were collected from China by A. David in 1869 and 1870, respectively. Four specimens [MNHN-IC-0000-5021 and 5022 (two), from the Yangtze River (or today’s Chang-Jiang); 7851 (one, from mountains of Jiangxi Province); and 7358 (one, from China, but without precise locality) were respectively collected by C. P. Dabry de Thiersant in 1863, 1868 and 1873. A comparison of X-ray photographs of two Chinese spiny eels reveals that *Sinobdellasinensis* has more vertebrae between the first and second anal spine pterygiophore than *Mastacembelusarmatus* (four or five vs. one or two) (Fig. 8). In terms of our examination on the X-ray photographs available in MNHN, the first and second pterygiophore of anal spines are separated by one or two vertebrae in two specimens (MNHN-IC-0000-5021 and 5022) (Fig. 9b, c), so indicating their misidentification of *M.armatus*. The specimen (MNHN-IC-0000-7490) is not the type of *S.sinensis* as it was collected in 1870, the same year as the publication of its original account, but slightly after the collection time (1868) of the type specimen of this species (see explanation below). The possibility is ruled out that the specimen (MNHN-IC-0000-7358) is the type of *S.sinensis* as it was caught in 1873, well after the publication of its original description. It is probable that the type of this species is amongst those caught by A. David (MNHN-IC-0000-5230 and 5573) and C. P. Dabry de Thiersant (MNHN-IC-0000-7851) in 1868. G.E. Simon is not the collector as spiny eels are not found in Chinese fish specimens, available in the MNHN collection, caught by him.

During 1868 to 1870, A. David made the second trip to central and western China for specimen collection (Luo 2005). His collection locations were in the Chang-Jiang Basin, including Zhenjiang and Jiangsu Province, Kiu-Kiang (or Jiujiang) and Lake Po Yang (or Poyang) of Kiang-Si (or Jiangxi) Province, Shanghai, Hankou and Yichang of Hubei Province, Chongqing and Chengdu of Sichuan Province; all collected specimens were sent to MNHN. Undoubtedly, six specimens of Chinese spiny eel (MNHN-IC-0000-5230 and 5573) were caught by A. David in the Chang-Jiang Basin. Information available in MNHN indicates that C.P. Dabry de Thiersant made a fish collection in the Chang-Jiang and mountain rivers of Jiangxi Province during 1868 to 1869. Based on fish specimens collected by him, Bleeker subsequently described many new Chinese species (Bleeker 1870a, Bleeker 1870b, Bleeker 1870c, Bleeker 1870d, Bleeker 1871, Bleeker 1872, Bleeker 1874). It cannot exclude the possibility that the specimen (MNHN-IC-0000-7851), collected in 1868 by Dabry de Thiersant from mountainous rivers of Jiangxi Province, was used as the type by Bleeker (1870a) to describe *Rhynchobdellasinensis* (now *Sinobdellasinensis*). No matter who is the collector, it is for certain that the type of this spiny eel is from the Chang-Jiang Basin. The specimen (MNHN-IC-0000-7851) from the mountain streams of Jiangxi Province is most likely the type as it agrees with the original description in, amongst others, body size.

Chinese spiny eels under the name of *S.sinensis* in the BMNH collection are also amongst mastacemblids examined by both Travers (1984a), Travers (1984b) and Vreven (2005). Three specimens (BMNH 1888.3.23: 60-62) of 119-169 mm TL and one specimen (BMNH 1895.5.31: 13-14) of 202 mm TL, which were donated by F. W. Styan, according to information available in this Museum, are from Kiu-Kiang (= Jiujiang City), Jiangxi Province (in the lower Chang-Jiang Basin) and in Shanghai (in the estuary of this river), respectively. Although three specimens BMNH 1888.3.23:60-62 were listed as types (Travers 1984a) or syntypes (Vreven 2005), they are unlikely the types of *S.sinensis* as they disagree with its original description in body size, far less than 199 mm TL of the type, as well as the collection time as indicated by their catalogued number, well after the publication of its original description. The specimen BMNH 1895.5.31:13-14 was listed as a syntype with a question mark (Vreven 2005), possibly due to its similar body size, close to 199 mm TL given in the original description for the type. It is not the type of this species because of its later collection time, as indicated by its catalogued number.

The discovery of this new species from the middle Zhu-Jiang Basin in Guangxi Province necessitates a re-diagnosis of *S.sinensis* and a delineation of its distribution in China. Characters typical for *S.sinensis* are given in the diagnosis. According to these characters, this species is extensively known from the mid-lower Chang-Jiang Basin. Populations of river basins north of this river, such as the Huai-He, Hai-He and Luan-He are conspecific with the species, as evidenced by molecular data (Fig. 7). This spiny eel is also found in the Li-Jiang and Luoqing-Jiang, two stream tributaries of the middle Xi-Jiang of the Zhu-Jiang Basin. The plausible explanation for this is that the spiny eel possibly dispersed into the two rivers from the Xiang-Jiang via the Ling Canal built in 214 BC.

Except for the count of vertebrae (Table 3), no distinct morphological and genetic variations are found between populations of the middle Zhu-Jiang and mid-lower Chang-Jiang Basin for *S.sinensis*. Sixteen specimens examined from Lingchuan County and Guilin City (in the Li-Jiang) and Yongfu County (in the Luoqing-Jiang) had 76-77 (mean 76.42) vertebrae, slightly fewer than 77-79 (mean 78) of four specimens examined from nearby Xing’an County (in the Xiang-Jiang, Guangxi Province; 76-78 (mean 77.6) of ten specimens from the Xiang-Jiang and Yuan-Jiang, two streams of Lake Dongting in Hengdong and Mayang Counties, Hunan Province; and 76-81 (mean 79.11) of 21 specimens collected from the Han-Jiang, the largest tributary of the Chang-Jiang Basin, in Xiangyang City and She-Shui in Dawu County, Fu-He in Sui County and Huan-He in Xiaochang County, three streams on the northern bank of the middle Chang-Jiang mainstream in Hubei Province. This variation can be plausibly explained by Jordan’s rule that stipulates a geographical tendency for populations of the same fish species from higher latitudes to have an increased number of vertebral centra (Jordan 1891, McDowall 2007).

This phenomenon is also observed for *S.sinensis* in east China. For example, four specimens caught from the Qiantang-Jiang Basin in Changshan County, Zhejiang Province, possessed 76-77 (mean 76.3), fewer than 77-79 (mean 77.8) of nine specimens from the Gan-Jiang (in Poyang Lake system) in Wanzai and Yongxin Counties, Jiangxi Province and 78-81 (mean 79.9) of seven specimens from the lower Chang-Jiang Basin in Susong and Tongcheng Counties and 77-79 (mean 77.6) of five specimens from the Huai-He Basin in Shangcai County of Henan Province and Huoshan County of Anhui Province. It seems that marked variations in the mean number of vertebrae were found between sampling locations north and south of the latitude (30°N). The highest number of vertebrae, though, was not found in the northern-most sampling location in the Luan-He (Luanzhou City). Mean vertebrae count also showed a longitudinally increasing variation from the Qiantang-Jiang to the middle and lower Chang-Jiang Basin. Collectively, the number of vertebrae for *S.sinensis* varies geographically, probably in association with temperature amongst different regions.

*Mastacembelusdienbienensis* is a Vietnamese species firstly described by Nguyen (2005) from the Nam Nua system in Dien Bien Phu City, in the Mekong River Basin of Vietnam. Although Kottelat (2013) ascribed it to *Sinobdella*, this generic assignment remains indeterminacy as this genus is currently unknown from the Mekong River Basin. Endruweit (2014) retained this spiny eel in *Mastacembelus* and confirmed that its four type specimens have been lost in RIA 1(Research Institute for Aquaculture No. 1, Bac Ninh, Hanoi). For this reason, its validity and generic placement remain yet to be resolved, only if topotypical specimens become available. For the time being, this species is excluded from *Sinobdella*.

*Mastacembeluskobayashii* was firstly described by Oshima (1926) from Taiwan Island. This spiny eel is only known by two type specimens in the original description. No additional specimens have been collected so far; its validity has remained yet to be resolved. Taiwanese spiny eels were previously identified as *Macrognathusaculeatum* (Bloch, 1786) (=*Sinobdellasinensis*) (Tzeng 1986). It has been considered as a subspecies of *S.sinensis* in the annotated checklist and type catalogue of fish genera and species described from Taiwan by Ho and Shao (2011). This taxonomic treatment is followed in this study.

## Supplementary Material

XML Treatment for Sinobdella longitubulus

A0397B4B-3BAE-556A-BBA5-0F0062EFBFFC 10.3897/BDJ.12.e123990.suppl1 Supplementary material 1 Detailed information on specimens Data type phylogenetic Brief description Detailed information on specimens used in this study. The species with * means the samples available in GenBank. File: oo_1059063.xlsx https://binary.pensoft.net/file/1059063 Peng Shan, Guangyu Li, E Zhang

## Figures and Tables

**Figure 2a.**
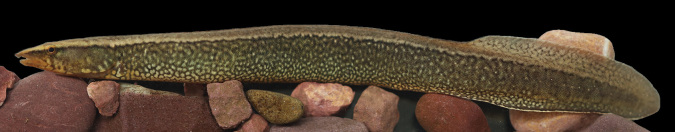
*Sinobdellalongitubulus*, IHB 202303066740, 148.7 mm SL, China: Guangxi Province: Guigang City: Pingnan County: Lilia Town: Datong-Jiang: Zhu-Jiang Basin;

**Figure 2b.**
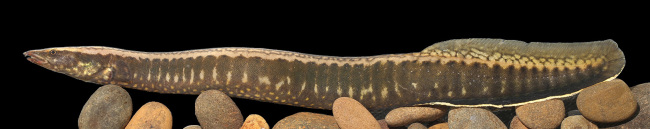
*Sinobdellasinensis*, IHB 202204062040, 183.2 mm SL, China: Hubei Province: Xiangyang City: Xiangzhou District: Han-Jiang: Chang-Jiang Basin.

**Figure 3a.**
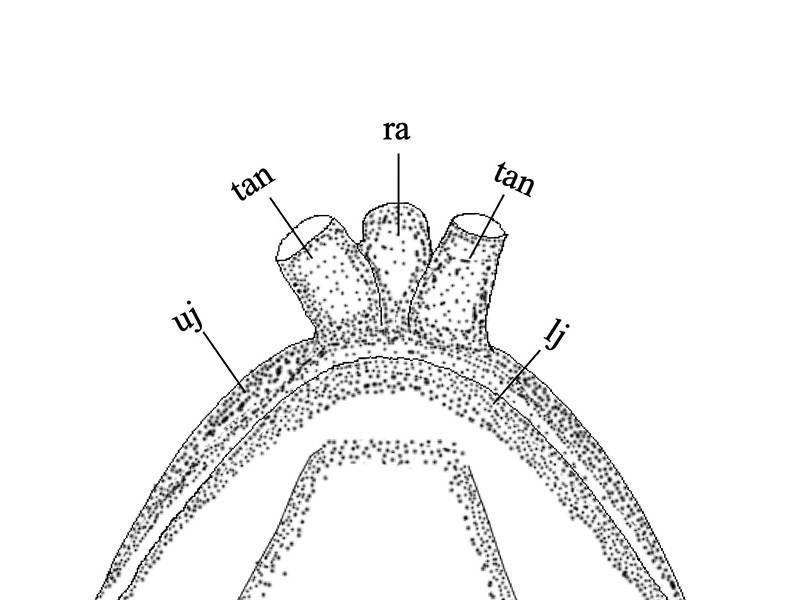
*Sinobdellalongitubulus*;

**Figure 3b.**
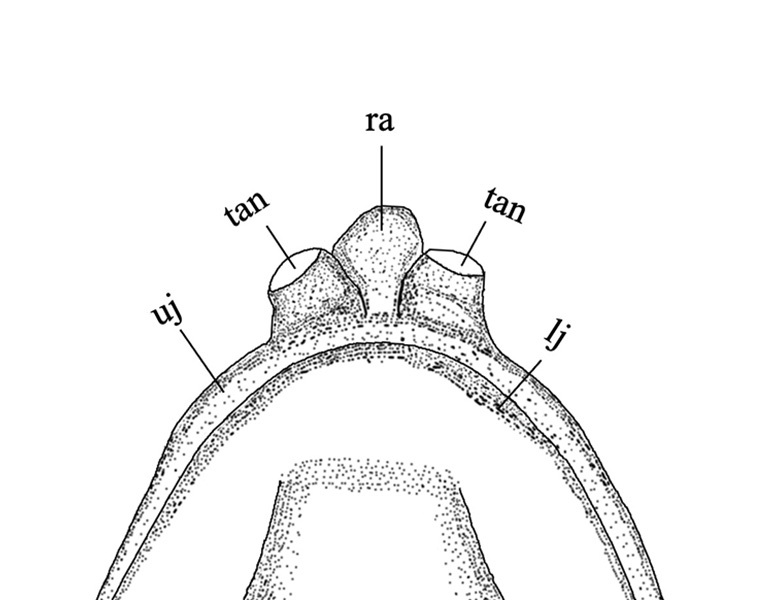
*Sinobdellasinensis*.

**Figure 8a.**
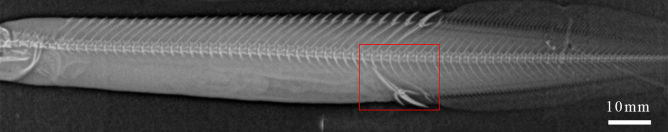
*Mastacembelusarmatus*, IHB 202010051228, caught from Li-Jiang of Xi-Jiang at Yangshuo County;

**Figure 8b.**
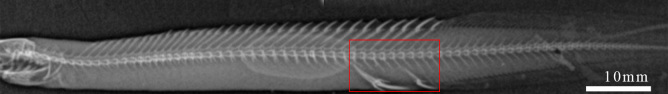
*Sinobdellasinensis*, IHB 202010051336, caught from Luoqing-Jiang of Xi-Jiang at Yongfu County, Guangxi Province.

**Figure 9a.**
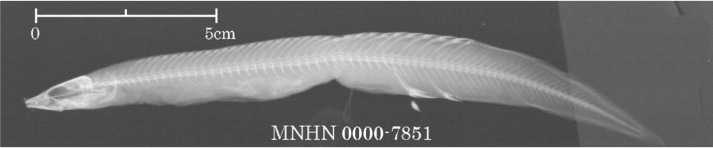
*Sinobdellasinensis* (MNHN-IC-0000-7851);

**Figure 9b.**
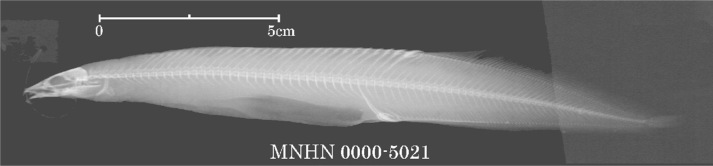
*Mastacembelusarmatus* (MNHN-IC-0000-5021);

**Figure 9c.**
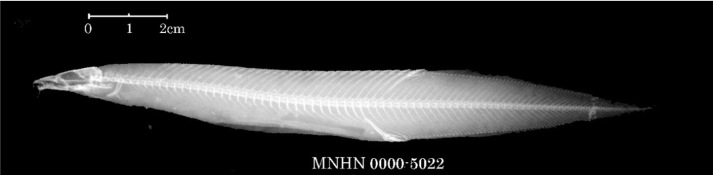
*Mastacembelusarmatus* (MNHN-IC-0000-5022).

**Table 1.** Morphometric data for two species of *Sinobdella* from China.

	***S.sinensis* (n = 32)**		***S.longitubulus***		
	Range	Mean±SD	**Holotype**	**Paratypes** (**n = 12)**	
				Range	Mean±SD
**Morphometric measurements**					
Standard length, SL (mm)	104.8-215.3	159.1±26.2	153.6	145.5-166.3	153.9±9.2
%**of SL**					
Head length	14.0-16.9	15.6±0.7	15.8	14.1-16.0	14.7±0.6
Body width	4.8-6.4	5.7±0.4	5.9	4.8-6.5	5.7±0.5
Body depth at anus	8.1-10.4	9.3±0.6	9.4	7.8-10.4	9.0±0.7
Pre-anal length	56.3-60.6	58.1±1.2	55.7	53.3-56.2	55.1±1.1
Post-anal length	39.4-44.9	42.0±1.3	44.3	41.3-44.6	43.6±1.0
Soft dorsal-fin base length	27.3-32.7	30.9±1.5	30.0	27.1-31.1	29.0±1.1
Soft anal-fin base length	28.5-34.0	32.0±1.5	33.2	30.1-33.2	31.6±1.0
Snout to first dorsal spine	17.7-23.3	20.1±1.2	21.9	18.7-22.1	20.2±1.1
Snout to last dorsal spine	66.5-72.0	69.0±1.4	71.1	66.8-72.5	70.1±1.8
Snout to first anal spine	58.2-64.0	60.8±1.5	58.4	56.1-59.9	58.7±1.2
Snout to last anal spine	64.9-71.9	68.3±1.5	67.4	65.2-68.5	67.1±1.1
Dorsal end of pectoral-fin base to last dorsal spine	51.8-56.0	54.3±1.0	55.1	52.4-57.6	55.2±1.5
First dorsal spine to last dorsal spine	45.3-51.7	49.1±1.5	49.2	47.7-53.5	50.1±1.8
First anal spine to last anal spine	6.0-8.3	7.2±0.6	8.6	7.3-8.6	8.2±0.4
% **of HL**					
Snout length	25.2-30.1	27.3±1.3	25.0	23.3-26.2	24.7±0.9
Eye diameter	7.7-12.3	10.6±1.1	11.8	10.6-14.5	12.1±1.1
Minimum interorbital distance	7.4-12.5	9.6±1.1	11.9	10.1-13.7	12.0±1.3
Postjaw angle length	67.0-76.6	72.5±2.5	66.8	69.8-76.2	73.3±2.2
Postorbital length	59.3-67.3	63.4±2.1	64.4	59.3-66.2	64.2±1.9
Post preorbital spine length	62.8-76.4	68.9±2.9	68.4	64.8-71.5	67.1±2.3
Postorbital to preoperculum	24.4-30.3	27.2±1.4	30.4	25.1-30.5	28.4±1.9
Snout to preoperculum	57.9-67.7	63.2±2.5	65.5	56.1-68.9	64.7±3.7
Upper jaw length	24.1-31.8	28.4±1.6	31.6	26.3-31.9	29.0±1.8
Lower jaw length	19.9-27.0	24.3±1.6	27.9	21.6-28.3	25.0±2.1
Pectoral-fin length	18.0-26.2	22.3±2.2	24.4	21.8-25.4	23.6±1.2
Dorsal end of pectoral-fin base to first dorsal spine origin	22.9-35.4	29.8±3.3	32.3	28.1-38.6	34.3±3.5
**Meristic counts**	***S.sinensis* (n=57)**		***S.longitubulus* (n=10)**		
Abdominal vertebrae	34(13),35(25),36(19)		33	32(1),33(8)	
Vertebrae between first anal spine and last dorsal spine supporting pterygiophores	6(9),7(38),8(10)		8	8(8),9(1)	
Caudal vertebrae	40(2),41(5),42(15),43(14),44(10),45(8),46(2),47(1)		43	43(6),44(2),45(1)	
Vertebrae total	76(11),77(15),78(8),79(12),80(8),81(5)		76	76(7),77(2)	
Dorsal spines	31(4),32(13),33(19),34(21)		33	32(1),33(8)	
Dorsal-fin rays	54(2),55(1),56(3),57(2),58(6),59(7),60(7),61(8), 62(6),63(2),64(4),65(3),66(3),67(2),68(1)		55	51(1),52(2),53(1),54(1),55(1),56(1),57(1),58(1)	
Anal-fin rays	53(1),55(2),56(2),57(3),58(7),59(3),60(11),61(5), 62(6),63(5),64(4),65(2),66(1),67(2),68(1),69(2)		58	51(2),52(1),53(3),57(1),58(2)	

**Table 2.** Information on specimens of Chinese spiny eels available in MNHN.

Catalogued number (No. specimens)	Location	Collector	Collection Time
MNHN-IC-0000-5021 (one)	Yangtze River	C. P. Dabry de Thiersant	1863
MNHN-IC-0000-5022 (one)	Yangtze River	C. P. Dabry de Thiersant	1863
MNHN-IC-0000-5230 (two)	China	A. David	1868
MNHN-IC-0000-5573 (four)	China	A. David	1868
MNHN-IC-0000-7851 (one)	Mountains of Jiangxi Province	C. P. Dabry de Thiersant	1868
MNHN-IC-0000-7490 (one)	China	A. David	1870
MNHN-IC-0000-7358 (one)	China	C. P. Dabry de Thiersant	1873

**Table 3.** The number of total vertebrae for *S.sinensis* from different locations in China.

Basin	River	Sampling location	Specimens examined	Total vertebrae (mean)
Middle Zhu-Jiang Basin	Li-Jiang	Guiling City and Linchuan County	n = 2	76 (76.0)
	Luoqing-Jiang	Yongfu County	n = 14	76-77 (76.4)
Middle Chang-Jiang Basin	Han-Jiang	Xiangyang City	n = 11	77-80 (78.5)
	She-Shui	Dawu County	n = 2	80,81(80.5)
	Fu-He	Sui County	n = 4	77,77,80,81 (78.8)
	Huan-He	Xiaochang County	n = 3	76,79,79,81(78.8)
Poyang Lake system	Gan-jiang	Wanzai County	n = 3	77,77,79(77.7)
	Gan-Jiang	Yongxin County	n = 6	77-79(78.0)
Dongting Lake system	Xiang-Jiang	Hengdong County	n = 1	78(78.0)
	Xiang-Jiang	Xing'an County	n = 4	77,78,78,79(78.0)
	Yuan-Jiang	Mayang County	n = 5	76-77(76.8)
Lower Chang-Jiang	Mainstream	Susong County	n = 6	78-81(79.8)
	Mainstream	Tongcheng County	n = 1	80(80.0)
Coastal rivers of southeast China	Qiantang-Jiang	Changshan County	n = 4	76,76,76,77(76.3)
	Min-Jiang	Sanming City	n = 1	77(70.0)
Huai-He Basin	Huai-He	Shangcai County	n = 4	77,77,78,79(78.3)
	Huai-He	Huoshan County	n = 1	77(77.0)
Coastal rivers of north China	Luan-He	Luanzhou City	n = 5	76,77,79,80,80(78.4)
